# Social Exclusion and Suicidal Ideation: Analysis of the Bereaved Living Alone

**DOI:** 10.1093/geroni/igaa057.225

**Published:** 2020-12-16

**Authors:** Bomi Choi, Hey Jung Jun, Sun Ah Lee

**Affiliations:** Yonsei University, Seoul, Republic of Korea

## Abstract

The aim of this study is to explore the relationship between social exclusion and suicidal ideation among bereaved older people living alone. When people with a significant loss in the familial relationship are exposed to social exclusion, they likely experience poor mental health and suicidal ideation. Using the Korean Community Health Survey 2017, logistic regression model was applied to the bereaved older people living alone, 65 to 110 of age (N=14,659). Social exclusion was comprised of three network-based components: exclusion from relationships, social participation, and community. Relationship exclusion means the lack of contact with family, friends, and neighbors, respectively, at least once a week. Social participation exclusion refers to the lack of participation in a religious, socializing, and leisure activity, respectively, at least once a month. Community exclusion covers two indicators of trust in neighbors and perception of neighborhood reciprocity. Results showed that indicators of relationship, social participation, and community exclusion were associated with suicidal ideation. Bereaved, living-alone older people were likely to have suicidal ideation when they lack contact with neighbors (OR=1.13, p<.05), participation in the religious activity (OR=1.12, p<.05) and socializing activity (OR=1.20, p<.05), and trust in neighbors (OR=1.29, p<.001). The moderation analysis showed that exclusion from socializing activity was associated with suicidal ideation only among females. The results of the analyses imply that interventions that promote social participation could improve the mental health of the bereaved older adults living alone.

